# SCOPE-based target and pathway enrichment identifies the histamine H1 receptor as a regulator of exodosis

**DOI:** 10.3389/fphar.2026.1972662

**Published:** 2026-10-19

**Authors:** Abhijeet Kapoor, Keith J. Kelleher, Suzanne M. Underhill, Sankalp Jain, Darian Williams, Brandon K. Harvey, Mark J. Henderson

**Affiliations:** 1 Division of Preclinical Innovation, National Center for Advancing Translational Sciences, National Institutes of Health, Rockville, MD, United States; 2 Cellular Stress and Inflammation Section, National Institute on Drug Abuse, National Institutes of Health, Baltimore, MD, United States

**Keywords:** bioinformatics workflow, drug repurposing, ER stress and exodosis, high-throughput screening, mechanism of action, polypharmacology

## Abstract

**Introduction:**

Phenotypic screening enables discovery of small molecules without requiring predefined targets, but mechanistic interpretation remains challenging due to polypharmacology and pathway complexity.

**Methods:**

We developed SCOPE (Screening Compound Ontology for Pathway Enrichment), a KNIME-based computational framework that links compound-level screening data to annotated targets and pathways to facilitate generation of mechanistic hypotheses. SCOPE integrates multi-source target annotations and performs sequential target- and pathway-level statistical enrichment using the screened compound library as the background to prioritize shared mechanisms of action.

**Results:**

Applied to a previously established high-throughput screen for modulators of endoplasmic reticulum (ER)-stress induced secretion of ER resident proteins, a process known as exodosis, SCOPE identified calcium signaling as the most enriched KEGG pathway without prior biological context. Its target enrichment revealed several G protein-coupled receptors (GPCRs) linked to inositol 1,4,5-trisphosphate receptors (IP3Rs)-mediated calcium signaling, with widespread antagonism of these receptors among compounds that reduced exodosis, implicating this pathway in the regulation of exodosis. Notably, SCOPE uncovered a novel role for the histamine receptor HRH1 in exodosis, which was validated by RNAi knockdown, gene knockout, and pharmacological modulation, implicating HRH1 as a potential therapeutic target for modulating exodosis.

**Discussion:**

These results highlight SCOPE’s potential to prioritize experimentally testable targets and pathways from phenotypic screening data.

## 1 Introduction

Phenotypic screening is a powerful strategy in early drug discovery because it enables identification of active compounds in biologically relevant systems without requiring predefined molecular targets (Vincent et al., 2022; Cooper et al., 2017). This approach is particularly well suited to interrogate complex processes involving polypharmacology, pathway crosstalk, and poorly characterized molecular drivers (Moffat et al., 2017). Advances in high-throughput screening formats, assay multiplexing, automated microscopy, and integrative data analysis have further enhanced the scalability and precision of phenotypic approaches, accelerating both mechanism-agnostic drug discovery and the repurposing of approved compounds (Ryoo et al., 2024).

One such biological process that has been used for phenotypic interrogation is the regulation of the endoplasmic reticulum (ER) proteome, which governs essential functions such as protein folding, post-translational modification, calcium signaling, and stress response activation (Mekahli et al., 2011; Zou et al., 2015; Martinez et al., 2016; Plate et al., 2016; Henderson et al., 2021). The ER plays a critical role in maintaining protein homeostasis (proteostasis), and disturbances to its environment, particularly disruptions in calcium balance, can trigger the accumulation of misfolded proteins and initiate the unfolded protein response (UPR). In extreme cases, these disruptions lead to a recently described phenomenon known as exodosis: the stress-induced secretion of ER-resident proteins into the extracellular space (Trychta et al., 2018; Henderson et al., 2021). This non-canonical release bypasses KDEL-receptor-dependent retention mechanisms and includes the export of molecular chaperones and protein-modifying enzymes, which are typically confined to the ER lumen. Exodosis has been observed in models of neurodegeneration, hyperthermia, ischemia-reperfusion injury, diabetes, and certain cancers, conditions often characterized by persistent ER stress and calcium dysregulation (Xu et al., 2005; Henderson et al., 2021; Trychta and Harvey, 2022; Dossat et al., 2024). Despite its potential relevance to disease-associated ER dysfunction, the mechanisms driving exodosis remain poorly defined, and no therapies currently exist that directly target or modulate this cellular response. The identification of novel small-molecule modulators of exodosis, through phenotype-driven approaches, represents one strategy that could be used to uncover the biological pathways involved in maintaining ER proteome integrity under stress.

In a prior study, we created a *Gaussia* luciferase-based secreted ER calcium-modulated protein (GLuc SERCaMP) reporter, which enabled a high-throughput phenotypic screen to identify small molecules that inhibit calcium depletion-induced exodosis (Henderson et al., 2021). Among the top characterized hits was bromocriptine, a dopamine receptor agonist with established clinical use but a poorly understood mechanism in the context of ER stress. While this work confirmed the feasibility of phenotypic screening to uncover modulators of ER proteostasis, mechanistic interpretation of the full set of hit compounds was obscured by their chemical diversity and potential for polypharmacology. This challenge remains common in phenotypic screens, where individual compounds may engage multiple targets and different compounds can converge on shared signaling pathways. Consequently, there is a critical need for computational approaches that systematically connect screening hits to their known molecular pharmacology and help reveal shared targets and pathway-level relationships that may otherwise be difficult to recognize.

Several computational tools have been developed for enrichment analysis and *post hoc* interpretation of compound sets. Drug Set Enrichment Analysis (DSEA; Napolitano et al., 2016) leverages transcriptional perturbation signatures from the Connectivity Map to detect shared pathways modulated by groups of phenotypically similar compounds. DrugPattern (Huang et al., 2018), Drugmonizome (Kropiwnicki et al., 2021), and CSgator (Park et al., 2019) perform drug set enrichment analysis using curated annotations for drug-target interactions, pathways, diseases, and other drug-associated features. Path4Drug (Fuzi et al., 2021) integrates compound-target mappings with Reactome (Jassal et al., 2020) pathway annotations in a tissue-specific context, with a primary focus on drug safety and toxicity assessment. While these approaches provide valuable complementary strategies for interpreting compound sets, they typically perform enrichment against a predefined universe of reference compounds, which may not reflect the specific chemical and target composition of the library used in a specific phenotypic screen. For screening applications, accounting for the actual screened library may be particularly important because target representation and annotation density can vary substantially among compound collections.

To address this challenge, we developed SCOPE (Screening Compound Ontology for Pathway Enrichment), a KNIME-based computational framework for context-aware target and pathway prioritization from phenotypic screening datasets. SCOPE integrates curated compound-target interaction data from publicly available sources including ChEMBL (Zdrazil et al., 2024), IUPHAR (Harding et al., 2024), PubChem (Kim et al., 2023), PharmGKB (Whirl-Carrillo et al., 2021), and DrugBank (Knox et al., 2024) together with optional user-supplied gene annotations. The compound-target mapping is performed for both the hit compounds and for the entire screened library, allowing target enrichment to be evaluated relative to the experimental screening background. Targets enriched among the screening hits are subsequently subjected to functional enrichment using STRING (Szklarczyk et al., 2023) pathway annotations to identify associated biological processes and signaling cascades.

We applied SCOPE to a previously published phenotypic screen of 10,124 small molecules, from the NCATS bioactive collections, which identified compounds that inhibited ER calcium depletion-induced exodosis (Henderson et al., 2021). SCOPE identified enrichment of GPCR targets, including receptors linked to IP3R-mediated calcium signaling, implicating this axis of GPCR signaling in the exodosis response. SCOPE also helped uncover a previously unrecognized role for the histamine receptor HRH1 in exodosis, which we validated experimentally through RNAi knockdown, gene knockout, and pharmacological modulation with selective agonists and antagonists. Together, these findings illustrate how SCOPE-based integration of phenotypic screening data with known compound pharmacology can generate experimentally testable mechanistic hypotheses, identify previously unrecognized regulators of complex cellular responses, and support rational drug development.

## 2 Materials and methods

### 2.1 Screening Compound Ontology for Pathway Enrichment (SCOPE) pipeline

SCOPE is a computational framework that connects the pharmacologically diverse set of hits obtained in high-content screens (HCS) or high-throughput screens (HTS) to their annotated biological targets and pathways providing hypotheses regarding shared targets and pathways among screening hits. Figure 1 provides a graphical overview of different steps involved in the SCOPE workflow which is implemented in KNIME (Berthold et al., 2008; version 4.5.3). Below we describe briefly each step of the pipeline.

**FIGURE 1:**
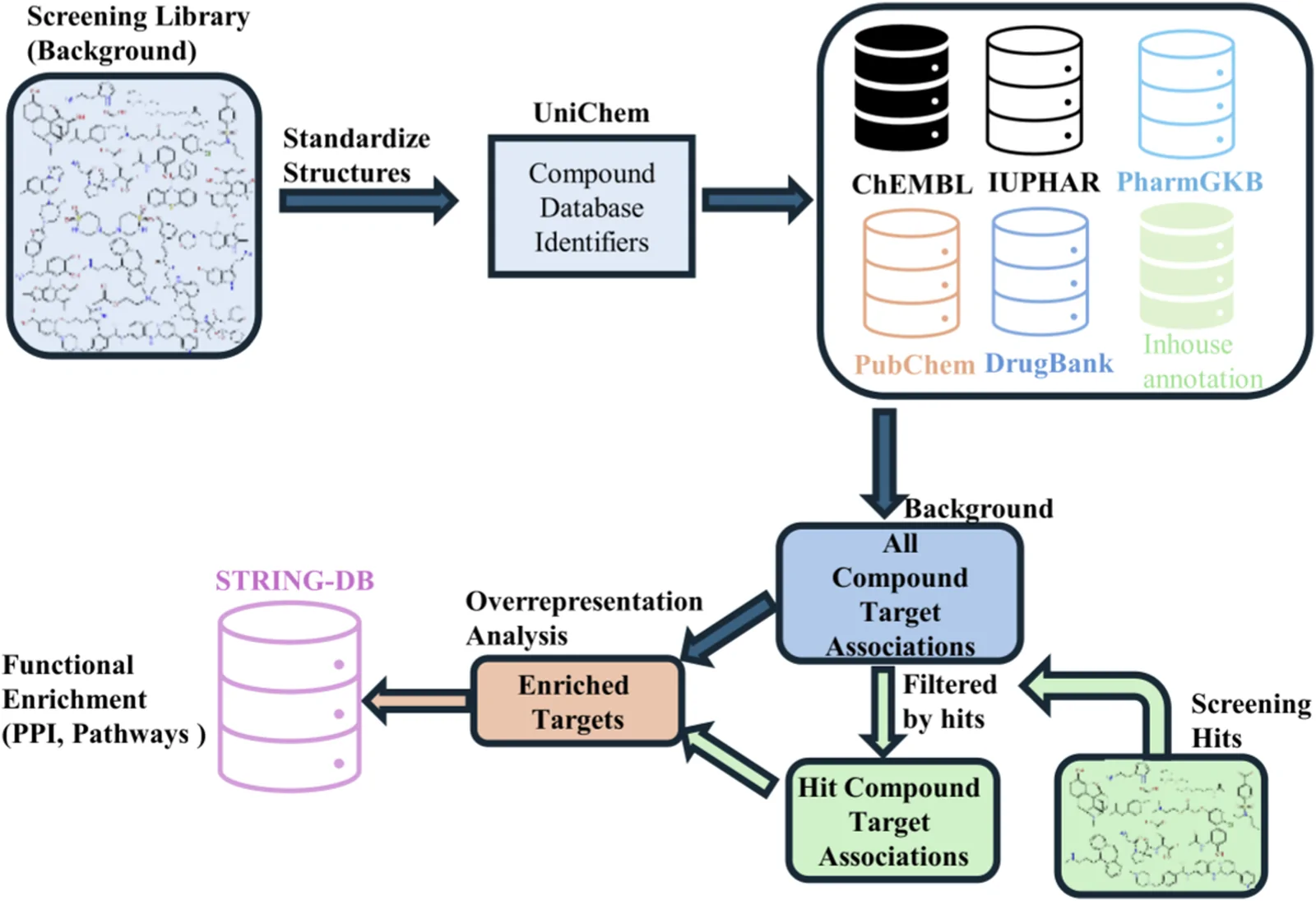
Schematic representation of the SCOPE workflow.

#### 2.1.1 Input data: using stabilizers of endoplasmic reticulum resident proteome as case study

The primary input to the workflow is an Excel file containing the compounds in the screening collection from which the hits were derived. Two columns, named: ‘SMILES’ and ‘Sample ID’, are required. Sample ID is a compound identifier associated with the SMILES representation of each input compound and it can be any string, such as a compound name or identifier. An optional ‘Gene Symbol’ column can also be provided to specify a single gene name known to be associated with each compound (Supplementary Table S1; see more details under data sources for compound-target interactions section). A second input Excel file is also required listing the ‘Sample ID’ of the compounds identified as screening hits (Supplementary Table S2). The Sample ID identifiers from this file are used to select the corresponding hit compound-target profiles from the complete set of compound-target profiles generated for the screening collection.

To demonstrate the application of the SCOPE workflow in interpreting hits resulting from a phenotypic screen we applied the workflow to compounds identified as stabilizers of the ER resident proteome (Henderson et al., 2021). Depletion of ER calcium can lead to the loss of resident proteins in a process termed exodosis. We previously performed a target-agnostic quantitative HTS using the GLuc SERCaMP assay, which monitors secretion of ER-resident proteins triggered by calcium depletion (Henderson et al., 2021). The assay was used to screen 10,124 compounds, including a collection of drugs approved by the FDA (US), EMA (EU), NHI (Japan), and HC (Canada) regulatory agencies. Compounds that decreased GLuc SERCaMP release, were subjected to counterscreens to exclude compounds that inhibited either secretory pathways, transgene expression, or cell viability resulting in the identification of 185 compounds that potentially stabilize the ER-resident proteome by different mechanisms. The 185 hits (Supplementary Table S2) along with the screening collection of 10,124 compounds (Supplementary Table S1) were used as inputs to the SCOPE workflow as described in the following sections.

#### 2.1.2 Compound standardization

Input compound structures were provided as SMILES strings and standardized using the RDKit structure normalizer node which applies a series of transformations (removal of hydrogens, stripping of salts, recombine charges, *etc.*) to standardize the input structures. The canonical SMILES of the corrected structure are then converted into InChIKey (IUPAC International Chemical Identifier Key) representation. For molecules containing salt, InChIKeys were generated both with and without salts to support flexible compound matching in downstream analyses.

#### 2.1.3 Generating compound-target profiles

Target profiles for each input compound are built by querying five different databases: PubChem (Kim et al., 2023), IUPHAR (Harding et al., 2024), PharmGKB (Whirl-Carrillo et al., 2021), ChEMBL (Zdrazil et al., 2024), and DrugBank (Knox et al., 2024). These databases either provide information on curated compound-target associations (such as DrugBank and PharmGKB) or contain experimentally derived target-associated bioactivity data (PubChem, IUPHAR, and ChEMBL). In the current implementation, only human proteins are considered as targets of interest. Furthermore, we apply a 10 µM cutoff for bioactivity-based target annotations. The target annotations obtained from the five databases can be supplemented with the optional user-provided gene annotation described above, allowing user-supplied annotations to be incorporated into the analysis. The metadata associated with target annotations from all resources are written out to CSV output files. To query these databases, we first use the UniChem (Chambers et al., 2013) web API to map the compound InChIKey to corresponding compound identifiers in the databases. Below we describe briefly the steps followed to generate target profiles from different sources:

*DrugBank*: The DrugBank database (https://go.drugbank.com/) is a comprehensive resource containing curated information about drugs and their targets. DrugBank provides access to its data in the form of XML file for noncommercial usage by obtaining the free academic license. Our workflow uses the drugbank id of the input compounds obtained through UniChem mapping to filter the XML file for relevant compounds and extract the target information. Any non-human target proteins and those with ‘unknown’ under known-action attribute (which provides information on whether the pharmacological action of the drug is due to a specific target interaction) are filtered out.

*PharmGKB*: Pharmacogenomics Knowledgebase (PharmGKB) database (https://www.pharmgkb.org) contains data about genes, drugs, diseases, and the pharmacogenomic relationships between them. Relationship files were downloaded from the database and PharmGKB compound id mapped by UniChem was used to parse the file for compound-target associations. Pharos (Kelleher et al., 2023; https://pharos.nih.gov/), an interface to the Target Central Resource Database (TCRD; https://habanero.health.unm.edu/tcrd/) which curates information about human targets, was used to map the gene symbols retrieved from the relationship file to UniProt accession number.

*IUPHAR*: IUPHAR/BPS Guide to Pharmacology database (https://www.guidetopharmacology.org/) is an expert-curated resource of ligand-activity-target relationships and it offers access to its data as JSON files through web services. UniChem mapped IUPHAR id of the input compounds are first used to retrieve the interactions data from the database which is then parsed to remove activity data above 10 µM and those against non-human targets. The target ids obtained is further parsed to retrieve the gene symbol. The UniProt accession numbers of target genes are then queried *via* Pharos.

*PubChem*: PubChem (https://pubchem.ncbi.nlm.nih.gov/) is an open chemistry database that collects information from over 500 data sources providing access to various types of chemical information including compound biological activities (Kim et al., 2023). PubChem Structured Data Query (SDQ) agent was used to retrieve structured-machine readable data by submitting queries as HTTP URLs. The UniChem mapped PubChem compound identifiers are used to generate a SDQ query to retrieve all the bioactivity data associated with human targets for each input compounds. The data is then parsed to retrieve target information removing any activity data above 10 µM.

*ChEMBL*: ChEMBL (https://www.ebi.ac.uk/chembl/) is a manually curated database of drug-like molecules with bioactivity data extracted from published literature, deposited datasets, and patents (Zdrazil et al., 2024). Compound activity data from ChEMBL was accessed *via* MySQL queries to an instance of ChEMBL version 33.0. For each input compound, all human target associated bioactivity records corresponding to binding and functional assays (or patents) with a target type of single protein and a pChEMBL value of five or above are extracted. pChEMBL is defined as: −log_10_ (molar IC50, XC50, EC50, AC50, Ki, Kd or Potency).

*Input gene symbol*: Pharos is used to map the optional user-provided gene symbol associated with each input compound to its corresponding UniProt accession number. This part of the workflow is optional and will be ignored in case the user inputs a blank column under ‘Gene Symbol’. For our input, we used the primary target annotations from our in-house database.

#### 2.1.4 Collating targets information from all sources

The compound-target profiles generated from different sources are combined, removing any duplicate compound-target pairs, and the resulting data are processed to generate three tables. The first output table contains a list of all unique targets along with columns indicating which sources the target annotation was derived from. The second table contains the compound-target pairs for all the compounds with resolved targets and is used for enrichment analysis (see next section). Compounds for which no target annotations were retrieved do not contribute to the target-enrichment analysis. To assess whether compounds with and without resolved target annotations occupied distinct regions of chemical space, compound structures were represented using chirality-aware Morgan fingerprints (radius 2, 2,048 bits) generated with RDKit (version 2025.9.2). To analyze dataset overlap and structural diversity, the resulting high-dimensional molecular fingerprints were projected onto a two-dimensional space using the UMAP algorithm implemented in the umap-learn library (version 0.5.12), using Jaccard distance and setting the nearest-neighbor and minimum-distance parameters to 30 and 0.1, respectively. This nonlinear dimensionality reduction framework has been shown to provide strong neighborhood preservation for chemical-space visualization across multiple molecular descriptor representations (Orlov et al., 2025). The third table lists, for each compound, the number of targets associated with it from different sources. These tables and any unresolved compounds are written to CSV files.

#### 2.1.5 Target enrichment analysis

Depending on the number of hit compounds being analyzed, the compound-target mapping may retrieve several hundred unique targets. Gaining biological insight from such a long list of protein targets is a cumbersome task and may lead to investigator bias toward a hypothesis of interest. Moreover, not all targets in the list will be relevant to the underlying biology as the presence of a target in the hit list will be influenced by the distribution of targets for the entire screening collection (background targets) assayed. To identify targets that are enriched in the hit list compared to the background we carry out a statistical overrepresentation analysis (ORA) that analyzes whether compound associations with certain targets are overrepresented in our hit list. For each target, a contingency table (shown in Table 1) was constructed, and ORA was done using Fisher’s exact test. The p-values resulting from Fisher’s exact test were adjusted for multiple comparisons using the Benjamini–Hochberg procedure (Yekutieli and Benjamini, 1999).

**TABLE 1:** Contingency table for overrepresentation analysis. $N_{B}$ is the number of compound-target associations for the entire screening collection (background targets), $T_{i}^{c}$ is the number of compound-associations for target_i_ in background, $T_{i_{hl}}^{c}$ is the number of compound associations for target_i_ in the hit list, and $N_{hl}$ is the number of compound-target associations in the hit list.

Association category	In hit list	Not in hit list	Total
Targeti associations	$T_{i_{hl}}^{c}$	$T_{i}^{c}-T_{i_{hl}}^{c}$	$T_{i}^{c}$
Other targets associations	$N_{hl}-T_{i_{hl}}^{c}$	$N_{B}-T_{i}^{c}-\left(N_{hl}-T_{i_{hl}}^{c}\right)$	$N_{B}-T_{i}^{c}$
Total	$N_{hl}$	$N_{B}-N_{hl}$	$N_{B}$

#### 2.1.6 Functional enrichment analysis

In the final step of the workflow, the enriched targets are queried through STRING database (Szklarczyk et al., 2023) to fetch the protein-protein interaction networks and functional enrichment results, including associated KEGG pathways and Gene Ontology Biological Process (GO-BP) terms.

### 2.2 Code availability

The KNIME workflow developed and used in this study is publicly available on GitHub at: https://github.com/ncats/scope.

### 2.3 Activity based compound action classification

Following target enrichment, we analyzed compound-target activity data from PubChem, ChEMBL, and IUPHAR for the 55 hit compounds associated with the 26 enriched targets to characterize their reported actions at these targets. A compound was classified as an inhibitor if its reported activity against a given target included parameters such as *K*_*i*_, *pK*_*i*_, *INH*, or any inhibitory concentration values (e.g., IC_50_, IC_20_, IC_80_). Conversely, compounds with reported *EC* values (e.g., EC_50_, EC_20_) were classified as activators. Because *K*_*i*_ and *pK*_*i*_ values do not always reflect inhibitory action, we cross-referenced the ‘Action’ field from IUPHAR and the ‘Action Type’ field from ChEMBL, both of which provide mechanism of action information, to correct activity metric-based classifications when this information was available. If the reported activity metric was unrelated to activation or inhibition (e.g., *K*_*d*_, *K*_*b*_, *K*_*m*_, *K*_*a*_), the compound action was classified as inconclusive. Compounds classified as both activators and inhibitors for the same target were labeled as having a mixed annotation. Finally, if a compound-target association originated from curated databases such as DrugBank or PharmGKB without specific activity parameters, it was labeled as unknown.

### 2.4 SERCaMP assay

Stable SH-SY5Y cells expressing the GLuc SERCaMP (Henderson et al., 2021) were grown in 10% serum-containing DMEM based media, supplemented with 1% Penn/Strep. Cells were plated at a density of 5 × 10^4^ cells/well in a 96-well tissue culture treated plate. After 24 h, the media was exchanged for 1.5% serum-containing media. 24 h after that, designated T0, the cells were treated with 10 μM H1R antagonists or agonists. At 16 h, the cultures that were treated with the histamine antagonists were treated with 100 nM thapsigargin for an additional 4 h. 10 μL supernatant samples were collected at T16 and T20 h timepoints and transferred to an opaque-walled plate for luciferase activity evaluation. 100 μL of a 10 μM coelenterazine (Regis Technologies, Morton Grove, IL) in PBS solution was injected into each well by a BioTek Synergy Neo2 Hybrid Multimode Reader (Biotek Synergy II, Winooski, VT) and luminescence subsequently read. Assays were performed in three independent experiments, n ≥ 11 per experiment and assessed by a one-way ANOVA.

### 2.5 RNAseq

SH-SY5Y cells were plated at a concentration of 8.0 × 10^5^ cells/well in a 6 well plate and incubated overnight to adhere. Cells were treated with 200 nM thapsigargin for 4 hrs, lifted from the plate and centrifuged, medium was removed and pellets were snap frozen on dry ice for 30 minutes. RNA was harvested and RNA integrity was evaluated by RIN measurement. RNA samples were sent to Azenta Life Sciences to perform bulk RNA sequencing and analysis. Expression of genes: HRH1 (ENSG00000196639), HRH2 (ENSG00000113749), HRH3 (ENSG00000101180), HRH4 (ENSG00000134489) were assessed.

### 2.6 CRISPR-mediated HRH1 knockout

SH-SY5Y cells were edited at the HRH1 locus by EditCo using CRISPR-Cas9 and multiple guide RNAs. The guide RNA sequences targeting exon two were GUG​ACC​ACU​UGG​ACA​UGA​GC (chr3:11,259,304), CUA​UCU​GCU​UGG​UCA​CAG​UA (chr3:11,259,161), and UCG​GUG​GCG​GAC​UUG​AUC​GU (chr3:11,259,262). The resulting pool of edited cells achieved 100% knockout of the HRH1 gene, with 77.58% alleles showing a deletion of 143 nt, 11.87% alleles showing deletion of 101 nt, and 10.55% of alleles showing a deletion of 41 nt. Primers used for sequencing were Forward 5′-ACA​AGA​CCA​CTA​TGG​CCA​GC-3′ and Reverse 5′-GTG​CTG​GCC​ACA​TAG​TCC​AT-3’. SH-SY5Y WT or HRH1-KO cells were transduced with AAV2-retro-EF1a-GLuc-SERCaMP-ASARTDL at 1.10 × 10^11^ Vg/mL for 24 h in 10% BGS containing media, medium was exchanged to 1.5% BGS for 24 h, and then cells were treated with vehicle control or 2-pyridylethylamine for 16 h 10 μL of supernatant was collected and assessed for released GLuc. Assays were performed in three independent experiments, n ≥ 3 per experiment, and assessed by one-way ANOVA.

### 2.7 siRNA-mediated knockdown of HRH1

GLuc-SERCaMP cells (Henderson et al., 2014) were reverse transfected with 20 nM H1R Silencer Select siRNA (Invitrogen, S6902, s223879 and s6904) with Lipofectamine RNAiMax (Invitrogen, 13778075). Control conditions were transfected with a scrambled control siRNA (Ambion, 4390843). 24 h after transfection, the cells were moved from media containing 10% serum into media containing 1.5% serum. At 48 h post-transfection, the cells were treated with 10 µM 2-pyridylethylamine for 16 h. Secreted GLuc was measured at this time, as described previously. Assays were performed in three independent experiments, n ≥ 3 per experiment, and assessed by multiple paired *t*-tests.

### 2.8 SERCaMP matrix screen of histamine receptor modulators with thapsigargin

SH-SY5Y human neuroblastoma cells stably expressing GLuc-SERCaMP (SH-SY5Y-GLuc-SERCaMP) cells were seeded at 1,000 cells per well (5 μL volume) in 1,536 well white tissue culture treated plates (Corning, Cat #7464) in DMEM-high glucose-sodium pyruvate (ThermoFisher Scientific, Cat #10569) supplemented with 10% bovine growth serum (Hyclone), 10 U/mL penicillin (GIBCO), 10 μg/mL streptomycin (GIBCO), and 20 mM HEPES. Cells were incubated for 5 h at 37 °C in a humidified incubator containing 5% CO_2_. Histamine antagonists (Rupatadine, Azelastine, and Clemastine) were added to cells using an Echo acoustic transferring machine (10 nL, 9 pt, 2× dilution; ranging from 20 μM to 78 nM) or neutral control (10 nL DMSO). Cells were incubated for 16 h (37 °C, 5% CO_2_) and thapsigargin was added in cross-titration (10 nL, 9 pt, 2× dilution; ranging from 1 μM to 3.9 nM) to deplete ER calcium stores in matrix format. Each drug concentration combination had four replicates. Cells were incubated for 4 h (37 °C, 5% CO_2_) and Gaussia luciferase activity in the medium was measured by adding 1 μL/well of 0.5× coelenterazine (final concentration 0.07×) prepared in Gaussia Luciferase Glow Assay Buffer (Pierce), without addition of the Cell Lysis Buffer Reagent. To measure cell viability, 1 μL/well of CellTiter-Glo reagent was added to replicate matrix plates in place of coelenterazine. Luminescence was measured using a ViewLux high-throughput CCD imaging system (Perkin Elmer) equipped with clear filters. GLuc-SERCaMP response values were normalized such that 0% response corresponds to DMSO alone and 100% to median of top 1% of compound wells. Compound synergy was evaluated based on the Bliss independence model and deviation from Bliss model (ΔBliss) was used to quantify the extent of synergy. In general, positive and negative delta Bliss scores in the synergy matrix indicate antagonistic and synergistic interactions between drugs, respectively. Statistical significance of ΔBliss interactions was assessed by 10,000 nonparametric bootstrap resamples (with replacement) of the replicate-level response data. For each iteration, the ΔBliss matrix was recalculated and the mean ΔBliss across combination cells was obtained. Interactions were considered significant when the 95% bootstrap confidence interval for the mean ΔBliss excluded zero, the Bliss-independence expectation. The 20 µM concentrations of azelastine and clemastine were excluded because of reduced cell viability.

## 3 Results

### 3.1 Target enrichment of screening hits identified as modulators of exodosis

To identify small-molecule modulators of ER exodosis, we previously carried out quantitative HTS of 10,124 compounds which led to the identification of 185 compounds that reduced thapsigargin-induced exodosis, which were interpreted to function through diverse mechanisms of action (Henderson et al., 2021). However, the pharmacologically diverse array of hits alone did not provide a clear picture of the underlying targets or mechanisms of action. Here, we took a data-centric approach, using the 185 compounds hit list together with the entire screening collection as inputs to our SCOPE workflow (Figure 1; Supplementary Tables S1 and S2; see methods for details) to systematically connect hits from the phenotypic screen to biological targets and pathways.

Running the SCOPE workflow on the entire screening collection (10,124 compounds) resulted in compound-target profiles for 6,829 compounds while no target association data was retrieved for remaining 3,295 compounds. A total of 44,771 compound-target pairs were obtained for the 6,829 compounds resulting in association with 2,702 unique targets. For the 185 compounds in the hit list, compound-target profiles were obtained for 143 hits, with 1,331 compound-target pairs resulting in 392 unique targets. To determine whether target annotation coverage was restricted to particular regions of the screening library chemical space, we compared compounds with and without resolved target annotations using Morgan fingerprint-based UMAP. Annotated and unannotated compounds showed extensive overlap with no clear global segregation by annotation status (Supplementary Figure S1), indicating that compounds with resolved target annotations broadly span the chemical diversity represented in the screening collection.

A target enrichment analysis comparing the compound-target profiles of the hit list with those of the background identified 26 targets enriched at 5% FDR and 70 targets enriched at 5% p-value (Supplementary Table S3). To assess how the screened-library background influences target enrichment, we compared target association frequencies among the screening hits with those in the complete screening library (Supplementary Figure S2A; Supplementary Table S3). CYP1A2 and CYP3A4 were the two most frequently associated targets among the screening hits, with 43 and 40 associated hits, respectively. However, these targets were also highly represented in the screening background, with 1,827 and 1,507 associated compounds, respectively, and neither was significantly enriched at the 5% FDR cutoff. More generally, targets that were frequently associated with hits but similarly represented in the screening library were not preferentially enriched, demonstrating how accounting for the screened-library background can substantially influence target prioritization.

A functional enrichment analysis of the entire set of 392 mapped targets, conducted using the STRING database, identified 170 enriched KEGG pathways, with various metabolism- and addiction-related pathways among the top ten, while the calcium signaling pathway ranked much lower (Figure 2A). In contrast, functional enrichment analysis using the 26 enriched targets identified by SCOPE (FDR <5%) revealed only 11 enriched KEGG pathways, with calcium signaling emerging as the top-ranked pathway (Figure 2B). Comparison of pathway ranks before and after target enrichment further demonstrated this reprioritization, with calcium signaling shifting from rank 18 among pathways identified using all mapped targets to rank 1 following target enrichment (Supplementary Figure S2B). Notably, an analysis of the 70 enriched targets, identified by a 5% p-value cutoff, still highlighted the calcium signaling pathway as the top-ranked pathway among 17 enriched KEGG pathways (Figure 2C), supporting the robustness of calcium signaling as a prioritized pathway across target-enrichment thresholds. STRING GO-BP enrichment analysis of the 26 FDR-enriched targets identified 232 enriched biological processes, including terms related to phospholipase C-activating GPCR signaling, response to histamine, regulation of cytosolic calcium, release of sequestered calcium into the cytosol, and cellular calcium homeostasis (Supplementary Table S4). These findings were consistent with the GPCR- and calcium-signaling themes highlighted by the KEGG analysis. Together, these results illustrate how the target-enrichment step can focus pathway-level interpretation and highlight biologically relevant signaling pathways without requiring prior knowledge of the screening hit’s mode of action.

**FIGURE 2:**
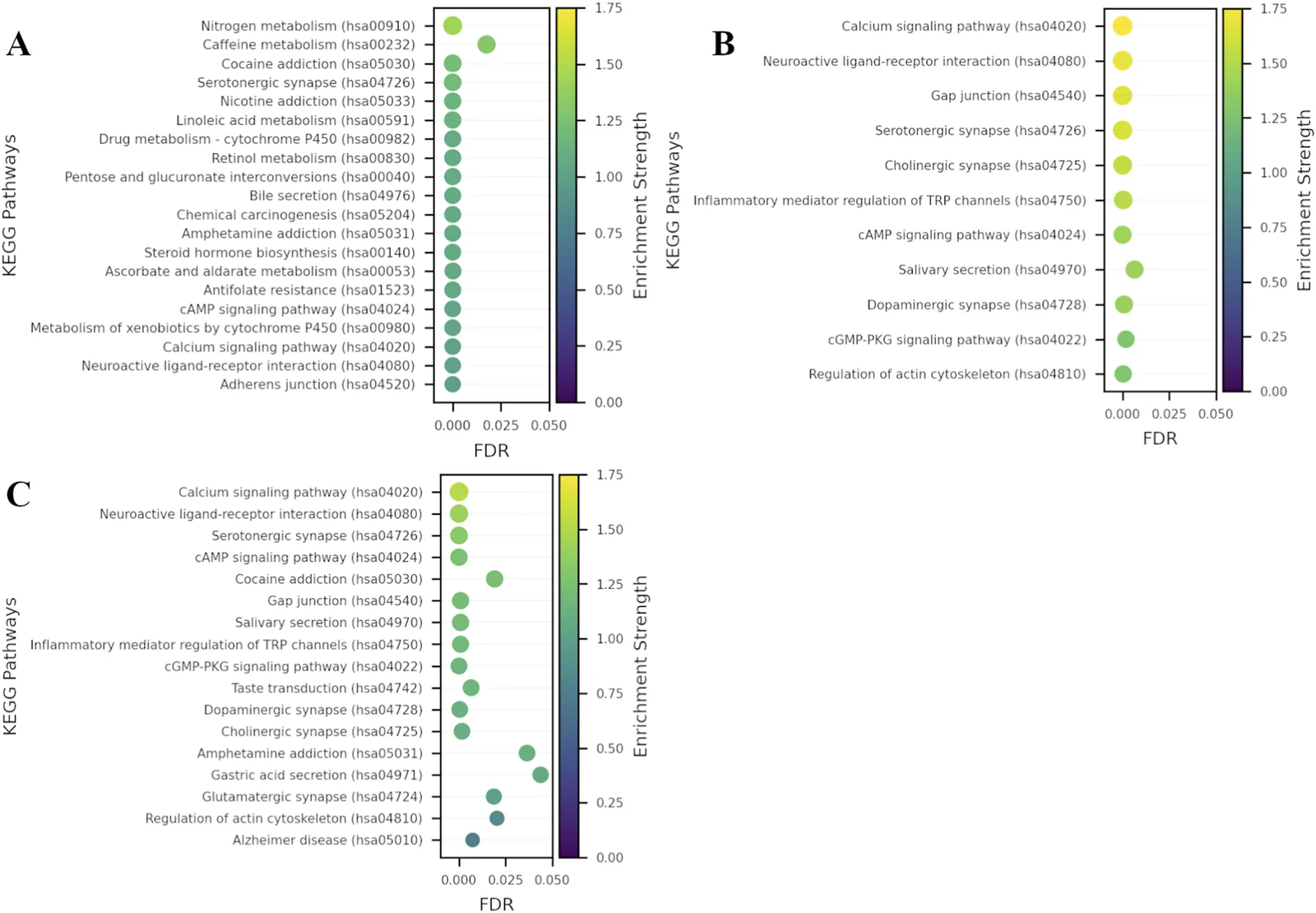
Enriched KEGG pathways identified by STRING functional enrichment analysis using **(A)** all 392 mapped targets for 185 screening hits, **(B)** 26 enriched targets identified by 5% FDR cutoff after target enrichment analysis, and **(C)** 70 enriched targets identified by 5% p-value cutoff after target enrichment analysis. The pathways are ranked by the enrichment strength metric from STRING, which quantifies the magnitude of enrichment as the base 10 logarithm of the ratio between the observed and expected number of proteins annotated with a given term.

### 3.2 Polypharmacology and GPCR-mediated regulation of ER exodosis

In our previous study, we prioritized five FDA-approved hits from the screen for further investigation. Dextromethorphan, bromocriptine, dantrolene, verapamil, and diltiazem were selected, due to their known roles in calcium modulation, with particular focus on characterizing bromocriptine (Henderson et al., 2021). Although bromocriptine is a dopamine D2 receptor (DRD2) agonist, our data suggested that bromocriptine’s anti-exodosis activity was unrelated to its dopaminergic activity leading us to suggest a non-dopamine receptor type GPCR, or more likely a distinct yet unknown target, as mediating bromocriptine’s ER proteome-stabilizing effect (Henderson et al., 2021). SCOPE analysis of compound-target action annotations revealed that bromocriptine exhibits a more nuanced polypharmacology wherein it has agonist annotations for a subset of dopamine and serotonin receptors and antagonist annotations for adrenergic and certain other dopamine and serotonin receptors (Supplementary Table S5). The analysis brings forward another possible explanation for bromocriptine’s effect on exodosis, in which a complex interplay of GPCR actions may contribute to the effect. This possibility was overlooked in our earlier interpretations of the data, as our efforts primarily focused on evaluating dopamine receptor modulation.

Among the 26 enriched targets, we observed prominent overrepresentation of GPCRs—particularly muscarinic (CHRM1-5), serotonin (HTR1A, HTR2A, HTR2B, HTR2C, HTR6), adrenergic (ADRA1A, ADRA1D, ADRA2A, ADRA2B), histamine (HRH1, HRH2), and dopamine (DRD1-3, DRD5) receptors (Figure 3). Several of these GPCRs couple to G_q_ proteins and activate the phospholipase C (PLC) and the IP3R signaling pathway resulting in the IP3 receptor mediated release of Ca^2+^ from the ER (Selway et al., 2012; Okubo, 2020; Mahmood et al., 2022). Based on this shared signaling axis, we hypothesized that hit compounds may attenuate exodosis by antagonizing signaling through the IP3R pathway. To further explore this hypothesis, we analyzed the compound-target activity data for hits that associated with the 26 enriched targets to classify compound actions at these targets (see methods for details). As shown in Figure 4, 55 out of 185 hits were functionally associated with one or more of the 26 enriched targets with several of the hits annotated as inhibitors, potentially functioning as pan-antagonists across multiple GPCRs (compound action ‘inhibitor’ highlighted in red circles in Figure 4), while for some other broadly acting compounds the mode of action could not be automatically assigned based on activity data alone (those labelled ‘inconclusive’). Notably, a subset of hits displayed more selective inhibitory annotation profiles for individual enriched targets, providing an opportunity to dissect more discrete regulatory roles for these targets. These included compounds such as NCGC00016366 (azacyclonol) which targets histamine receptor (HRH1), NCGC00024378 (lobeline) which targets HTR2A, NCGC00024788 (2-CMDO) which targets DRD2, NCGC00024880 (AR-C239) that inhibits ADRA1A, NCGC00025328 (SB-258585) that inhibits HTR6, and NCGC00025335 (esreboxetine) which targets SLC6A2. Notably, in addition to azacyclonol, several FDA-approved selective antihistamines also appeared in this list despite showing some off-target activity (Figure 4). These included ketotifen (NCGC00015580), cyclizine (NCGC00016421), clemastine (NCGC00016710), desloratadine (NCGC00159325), and chlorcyclizine (NCGC00179384). These findings prompted us to further investigate the role of histamine receptor signaling in modulating the exodosis response.

**FIGURE 3:**
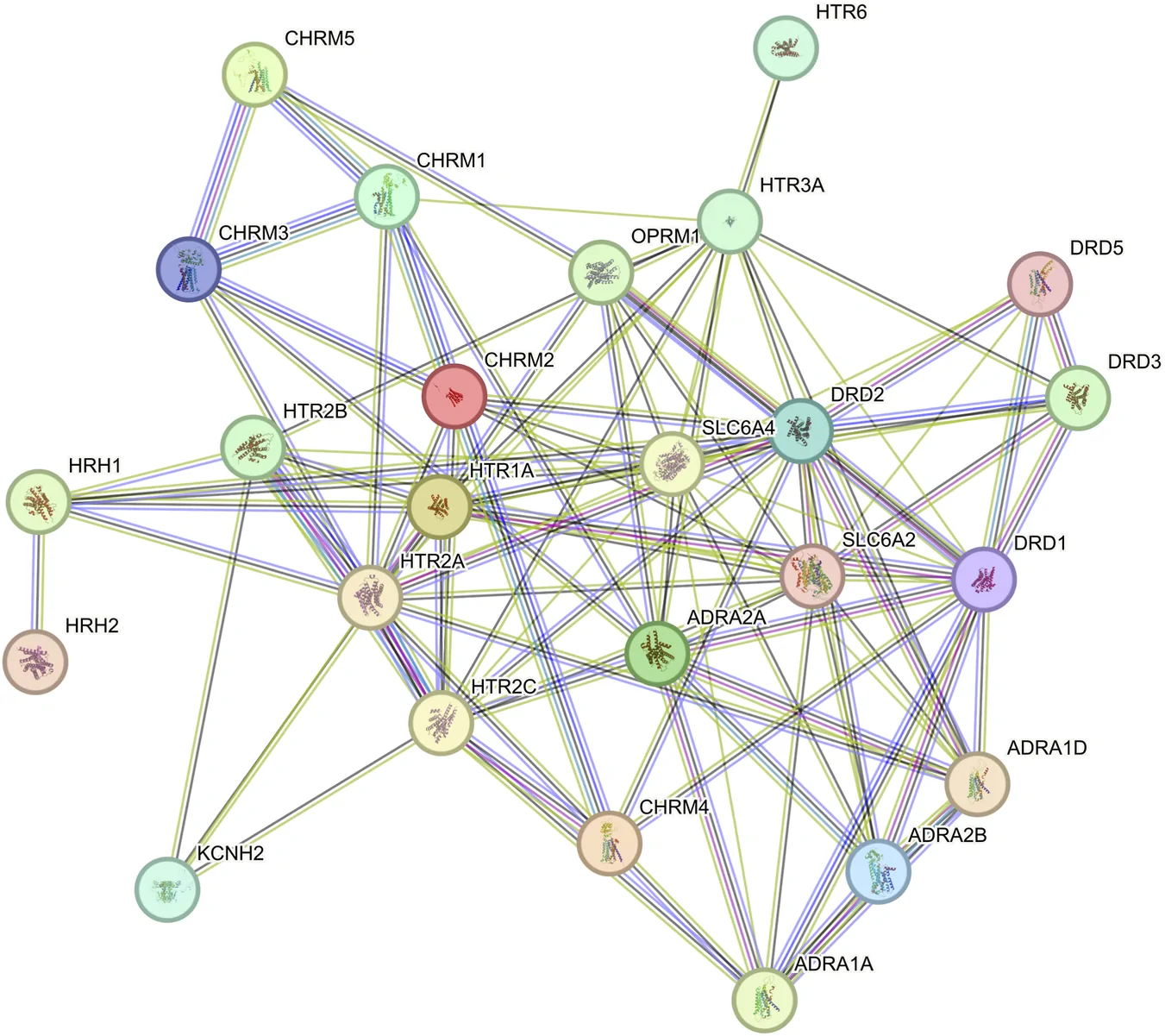
Protein-protein interaction network from STRING for the 26 enriched targets identified by 5% FDR cutoff after target enrichment analysis.

**FIGURE 4:**
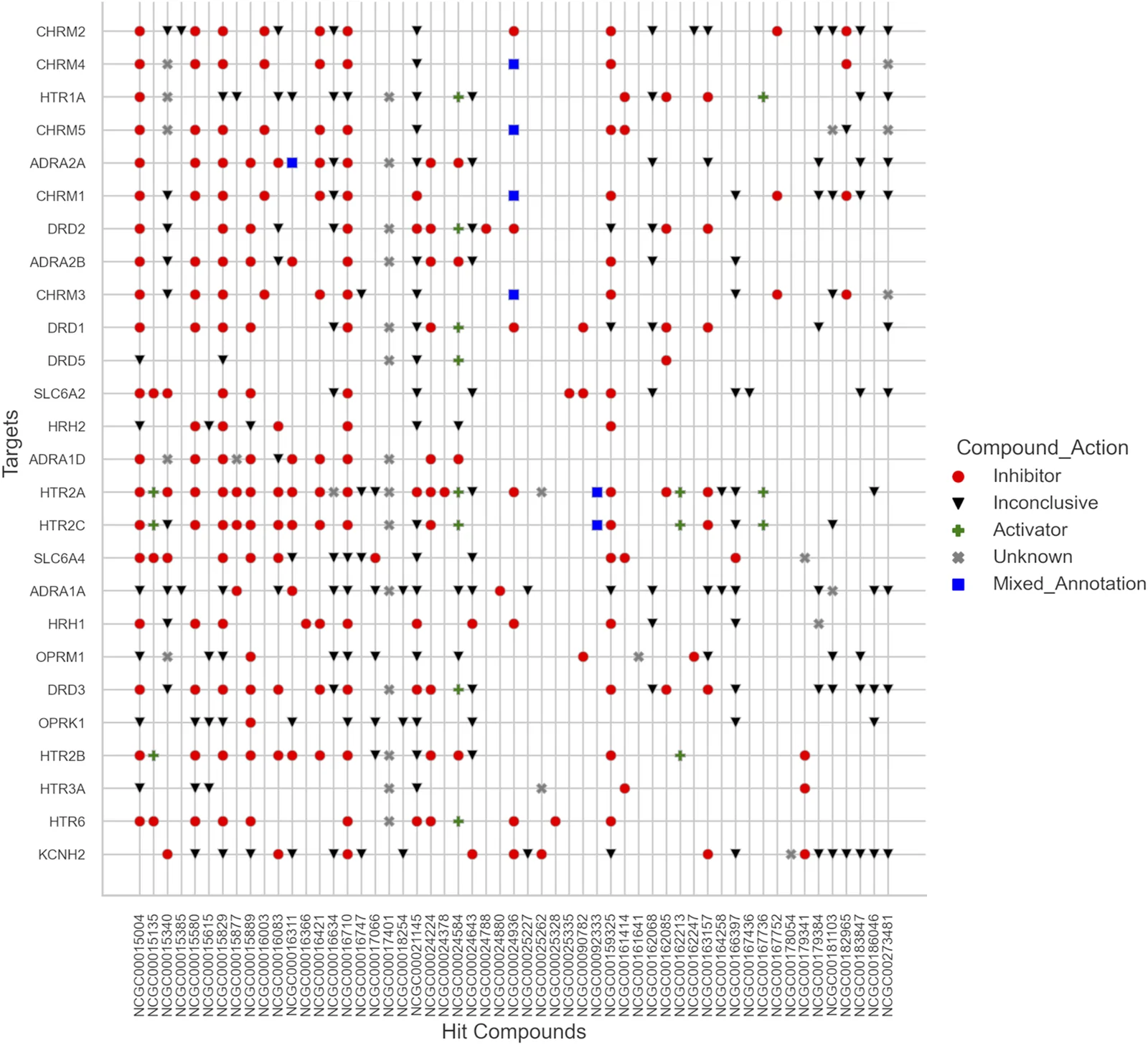
Classification of 55 hit compounds (NCGC IDs) action associated with the 26 enriched targets based on reported activity annotations. Compounds were classified as inhibitors if annotated with K_i_, pK_i_, INH, or inhibitory concentrations (e.g., IC_50_, IC_20_), and as activators if associated with EC values (e.g., EC_50_, EC_20_). Because K_i_ and pK_i_ values do not necessarily indicate inhibitory action, IUPHAR ‘Action’ and ChEMBL ‘Action Type’ annotations were used to refine classifications when available. Metrics unrelated to functional modulation (e.g., K_d_, K_b_, K_m_, K_a_) were labeled inconclusive. Compounds annotated as both activators and inhibitors for the same target were marked as mixed. Associations from curated sources (e.g., DrugBank, PharmGKB) without specific activity values were labeled unknown.

### 3.3 Histamine receptor silencing attenuates exodosis

To investigate the role of histamine signaling in exodosis, we utilized the previously described GLuc SERCaMP assay (Henderson et al., 2021) to monitor the exodosis response in SH-SY5Y human neuroblastoma cells. We first examined the expression of the histamine receptor paralogs in SH-SY5Y cells, which revealed appreciable expression of both HRH1 and HRH3, with near undetectable levels of HRH2 and HRH4 (Figure 5A). In the absence of thapsigargin-induced ER stress, selective activation of HRH1 using two selective agonists: *N-*methylhistaprodifen (NCGC00186016-01; Seifert et al., 2003) and 2-pyridylethylamine (NCGC00159581-01; Ratnala et al., 2004), resulted in an increase in GLuc SERCaMP signal indicating an increase in exodosis response (Figure 5B). To determine whether this effect is HRH1-dependent, we assessed agonist response in HRH1 knockout SH-SY5Y cells. While 2-pyridylethylamine significantly increased exodosis in wildtype cells, this effect was abolished in HRH1 knockout cells, demonstrating that agonist-induced enhancement of exodosis is mediated specifically through HRH1 (Figure 5C). In agreement, siRNA-mediated knockdown of HRH1 resulted in a significant reduction in exodosis response to histamine receptor agonist compared to wildtype and scrambled controls (Supplementary Figure S3), further supporting a functional role for HRH1 in regulating exodosis. We next evaluated whether pharmacological inhibition of HRH1 produces the opposite effect under ER stress conditions. We tested three selective histamine receptor antagonists: rupatadine (Shamizadeh et al., 2014; pIC_50_ 8.4), azelastine (Corren et al., 2005; pK_i_ 8.9; FDA approved), and clemastine (Dorsch et al., 1982; pK_i_ 10.3; FDA approved), and observed that all three compounds significantly reduced GLuc SERCaMP signal relative to control suggesting anti-exodosis activity (Figure 5D). To further characterize this effect, we performed concentration-matrix combination experiments with thapsigargin for each of the three HRH1 antagonists. Across a range of concentrations, all three antagonists consistently reduced GLuc SERCaMP response, and the ΔBliss analysis (deviation from the Bliss synergy model) revealed predominant antagonistic interactions (blue regions indicated by positive delta Bliss scores) across much of the matrix (Supplementary Figure S4), with mean ΔBliss values of 0.105 for rupatadine (95% bootstrap CI, 0.082–0.129), 0.151 for azelastine (95% CI, 0.129–0.176), and 0.148 for clemastine (95% CI, 0.125–0.172). These results indicate that HRH1 antagonists counteract thapsigargin-induced exodosis in a dose-dependent manner, further supporting a functional role for HRH1 in regulating the exodosis response.

**FIGURE 5:**
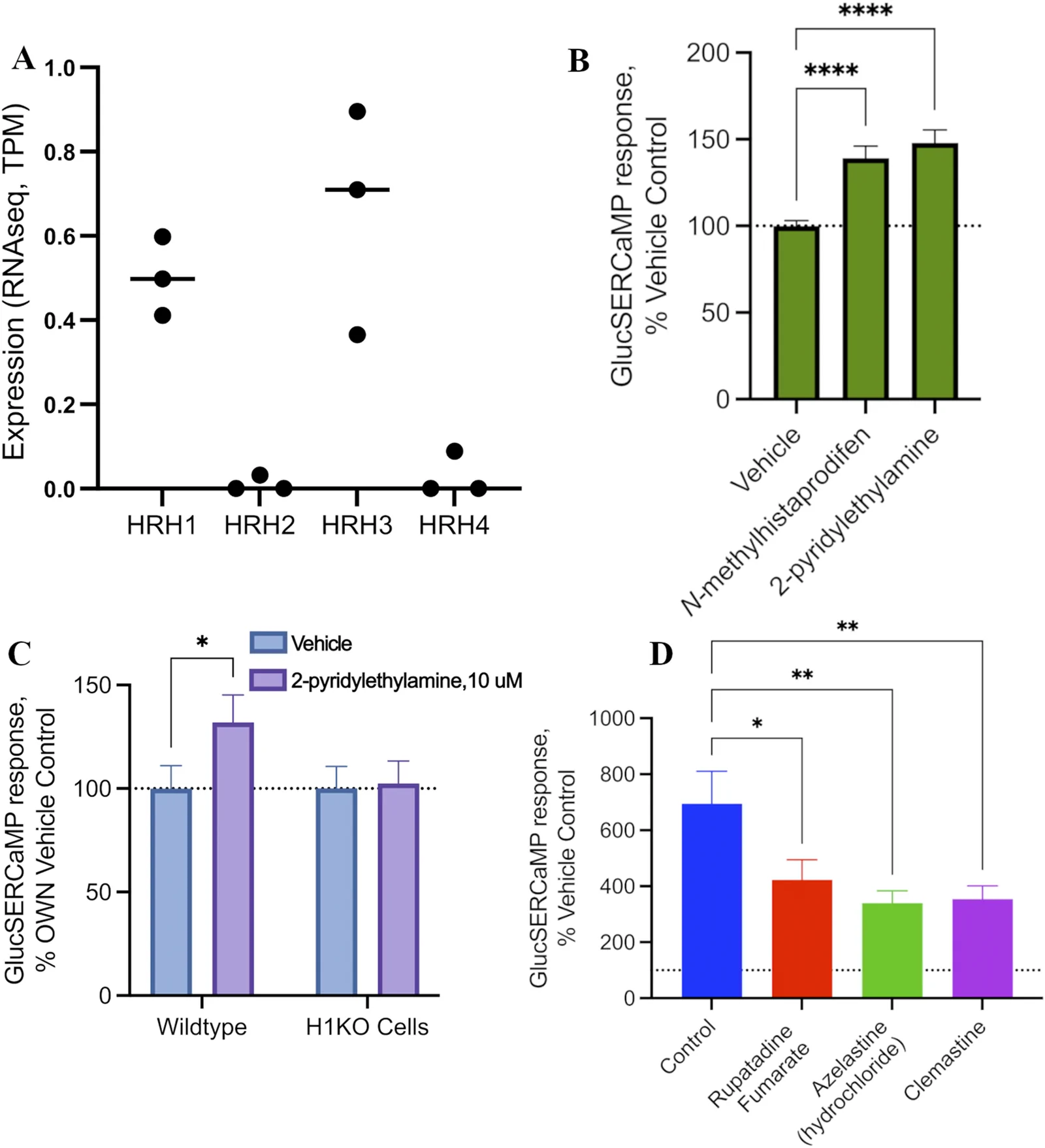
Effect of histamine receptor 1 (HRH1) on exodosis response. **(A)** RNAseq data examining the expression of the histamine receptor paralogs in SH-SY5Y cells. **(B)** Two different selective H1 receptor agonists (10 μM *N-*methylhistaprodifen or 10 μM 2-pyridylethylamine) were applied to stable GLuc SERCaMP SH-SY5Y cells for 16 h (three independent experiments, n ≥ 11 per experiment, *<0.05, **<0.01, ****<0.0001 by one-way ANOVA). **(C)** Wildtype and H1 knockout SH-SY5Y cells were transduced with AAV2-retro-EF1a-Gluc-SERCaMP-ASARTDL. After 48 h, the cells were treated with vehicle control or 10 μM 2-pyridylethylamine for 16 h. Wildtype cells released ∼30% more GLuc than their H1 knockout counterparts. Data normalized to own vehicle control (three independent experiments, n ≥ 3 per experiment, *<0.05 by multiple paired *t*-tests). **(D)** Stable GLuc SERCaMP SH-SY5Y cells were pre-treated with 10 μM of one of three selective H1 antagonists (rupatadine, azelastine, and clemastine) for 16 h. Subsequently, they were treated with 100 nM thapsigargin for 4 hours. Data here represents the results of three independent experiments (n = 3 replicates per experiment), all normalized to their own vehicle control condition (*<0.05, **<0.01, ****<0.0001 by one-way ANOVA).

## 4 Discussion

Phenotypic screening is valuable for probing complex biological processes without prior assumptions about molecular targets, particularly in multifactorial disorders where traditional target-based approaches may fall short. However, interpreting the results of such screens remains challenging due to the pharmacological and structural diversity of hits, the polypharmacology of small molecules, and the lack of mechanistic context linking compounds to pathways. Manual analysis of large (or even moderately sized) hit lists to gain biological insights can be a cumbersome task, often leading investigators to focus on a much smaller subset of hit compounds and prioritize results that align with prior hypotheses, thereby missing unexpected or novel relationships embedded in the dataset. To address these challenges, we developed the Screening Compound Ontology for Pathway Enrichment (SCOPE) platform, an extensible KNIME-based framework that integrates compound-target annotation with sequential target- and pathway-level enrichment to support generation and prioritization of mechanistic hypotheses from phenotypic screening hits. SCOPE leverages the specific target space represented in a given screening library to identify protein targets statistically overrepresented among phenotypic screening hits, followed by functional enrichment analysis to identify associated biological processes. This two-step approach, consisting of target-level enrichment followed by pathway-level inference, helps prioritize targets and pathways that may contribute to the observed phenotype while accounting for the target composition of the screened compound library.

In this study, we applied SCOPE to a target-agnostic high-throughput screen for stabilizers of the ER resident proteome (Henderson et al., 2021) to investigate potential mechanisms underlying compound activity. The hit compounds from this screen reduced secretion of GLuc SERCaMP, a reporter of the exodosis response induced by ER calcium depletion. While a broad functional enrichment analysis of all 392 mapped targets from the hit list identified numerous pathways, including metabolism- and addiction-related ones, functional enrichment of the 26 targets identified by SCOPE’s target enrichment analysis provided a more focused pathway profile, with calcium signaling emerging as the top enriched KEGG pathway. This comparison illustrates how restricting pathway analysis to targets enriched relative to the screened-library background can help focus pathway-level interpretation without prior knowledge of the compound’s mode of action. By applying target enrichment relative to the screened-library background, SCOPE deprioritized targets that were similarly represented among hits and the screening library, narrowing the analysis to candidate molecular regulators that may mediate ER exodosis and could be prioritized for experimental follow-up, thereby identifying putative mechanisms that would be difficult to discern using all mapped targets alone.

The prominent representation of GPCRs among the 26 enriched targets, including muscarinic, serotonin, adrenergic, histamine, and dopamine receptors, is particularly noteworthy and suggests that these receptors may serve as signaling hubs whose pharmacological modulation alters the cellular response to ER calcium depletion. Several of these GPCRs are known to couple to G_q_ proteins, activating the PLC and IP3R signaling pathway, which mediates Ca^2+^ release from the ER (Selway et al., 2012; Okubo, 2020; Mahmood et al., 2022). This shared signaling axis led us to hypothesize that many hit compounds might attenuate exodosis by antagonizing signaling through the IP3R pathway. Indeed, our activity-based classification showed that many hits associated with these GPCRs had inhibitory annotations across multiple receptors, potentially acting as pan-antagonists. For example, bromocriptine, a previously characterized hit (Henderson et al., 2021), has established clinical activities in the context of Parkinson’s and diabetes, but its relationship to ER stress and exodosis are incompletely understood. Our SCOPE analysis highlighted bromocriptine’s broad polypharmacology across several GPCRs, suggesting a more complex mechanism of action beyond its canonical dopamine D2 receptor agonism, and offering a plausible explanation for its anti-exodosis activity. Still, some hits displayed more selective inhibitory profiles for specific enriched targets, such as HRH1, HTR2A, DRD2, ADRA1A, HTR6, and SLC6A2.

We experimentally validated the functional relevance of one of these targets, HRH1, using selective small molecule antagonists and agonists. Selective antagonists of HRH1 (clemastine, rupatadine, and azelastine) consistently suppressed thapsigargin-induced secretion of GLuc SERCaMP, supporting a role for histaminergic signaling in modulating exodosis. In contrast, the HRH1 selective agonists (*N-*methylhistaprodifen and 2-pyridylethylamine) enhanced secretion, further strengthening the hypothesis that HRH1 activation potentiates the exodosis response. Furthermore, genetic reduction of HRH1 using RNAi-mediated knockdown or CRISPR-mediated knockout abrogated the response to agonists, providing genetic confirmation of HRH1’s role in mediating the exodosis phenotype. These findings are consistent with prior studies showing that HRH1 activity influences intracellular calcium homeostasis and ER stress (Deniaud et al., 2008; Chen et al., 2022; Fan et al., 2024). Although HRH1 has previously been linked to intracellular calcium signaling and ER stress, its role in regulating exodosis has not been established. Our results therefore identify HRH1 as a previously unrecognized modulator of exodosis and suggest that HRH1 may represent a potential target for pharmacological modulation of this response. Whether the effect of HRH1 on exodosis is mediated through the PLC/IP3R signaling axis remains to be determined.

Despite the novel insights into exodosis provided by the SCOPE framework, this study is not without limitations. First, while SCOPE integrates data from five major public databases, target association data could not be retrieved for 3,295 out of 10,124 compounds in the screening library, and 42 of the 185 hits lacked target annotations. This reflects the broader challenge of incomplete target annotation for many small molecules, especially investigational or less-characterized compounds. Because enrichment is necessarily restricted to compounds with available target annotations, incomplete annotation coverage may bias the analysis toward better-characterized compounds and targets. However, chemical space analysis of the screening library showed extensive overlap between annotated and unannotated compounds, indicating that the annotated subset broadly spans the chemical diversity represented in the screening collection rather than being confined to a distinct structural region. However, this structural coverage does not compensate for the absence of experimentally established target information for unannotated compounds. To improve coverage, future efforts could integrate additional annotation resources or employ predictive modeling to infer targets for compounds without known annotations. Second, SCOPE was evaluated here using a single phenotypic screening dataset. Although the present analyses demonstrate how the screened-library background influences target and pathway prioritization within this dataset, application to additional independent screens across different biological systems will be important to further establish the generalizability of the approach. Finally, the experimental validation of HRH1’s role in ER exodosis was primarily conducted in SH-SY5Y human neuroblastoma cells. While this cell line is relevant for neurodegeneration models, the findings may not be fully generalizable to other cell types or *in vivo* systems, which could exhibit different ER stress responses, histamine receptor expression, or histamine receptor signaling pathways. RNA-seq analysis also showed appreciable expression of HRH3 in SH-SY5Y cells. However, unlike HRH1 and HRH2, HRH3 was not among the FDR-enriched targets identified by SCOPE and was not functionally evaluated in the present study; whether HRH3 also contributes to regulation of exodosis therefore remains to be determined. Further experimental evaluation of HRH1 in additional biological models, as well as investigation of HRH3 and other enriched targets identified by SCOPE, will help define the broader contribution of these signaling pathways to exodosis.

In summary, SCOPE provides a flexible computational framework for generating and prioritizing mechanistic hypotheses from phenotypic screening data by integrating compound-target annotations with sequential target- and pathway-level enrichment. Its application to a screen for ER proteome stabilizers not only revealed novel regulators of exodosis, such as HRH1, but also illustrated how compound-target enrichment can help prioritize shared targets and pathways among polypharmacological screening hits. While demonstrated here in the context of ER proteostasis, we anticipate that SCOPE will be applicable to other complex phenotypic screening campaigns. Applying the pipeline to screens across diverse biological processes and disease areas will further establish its generalizability and utility in accelerating mechanism-agnostic drug discovery and drug repurposing. As phenotypic screening continues to grow in scale and complexity, tools like SCOPE may help bridge high-throughput discovery efforts with systems-level interpretation, advancing our ability to identify new mechanisms, therapeutic targets, and drug repurposing opportunities.

## Data Availability

The original contributions presented in the study are included in the article/Supplementary Material and are publicly available. The RNA-sequencing data can be found in the NCBI Gene Expression Omnibus (GEO) under accession number GSE349367.
