# Supplementary material for: SCOPE-based target and pathway enrichment identifies the histamine H1 receptor as a regulator of exodosis
Source: Front Pharmacol. 2026 Oct 19;17:1972662. doi: 10.3389/fphar.2026.1972662 (PMC13724830; doi:10.3389/fphar.2026.1972662)
Supplement: Supplementary file 1 [file Supplementaryfile2.pdf]

Supplementary Material

1.1 Supplementary Figures

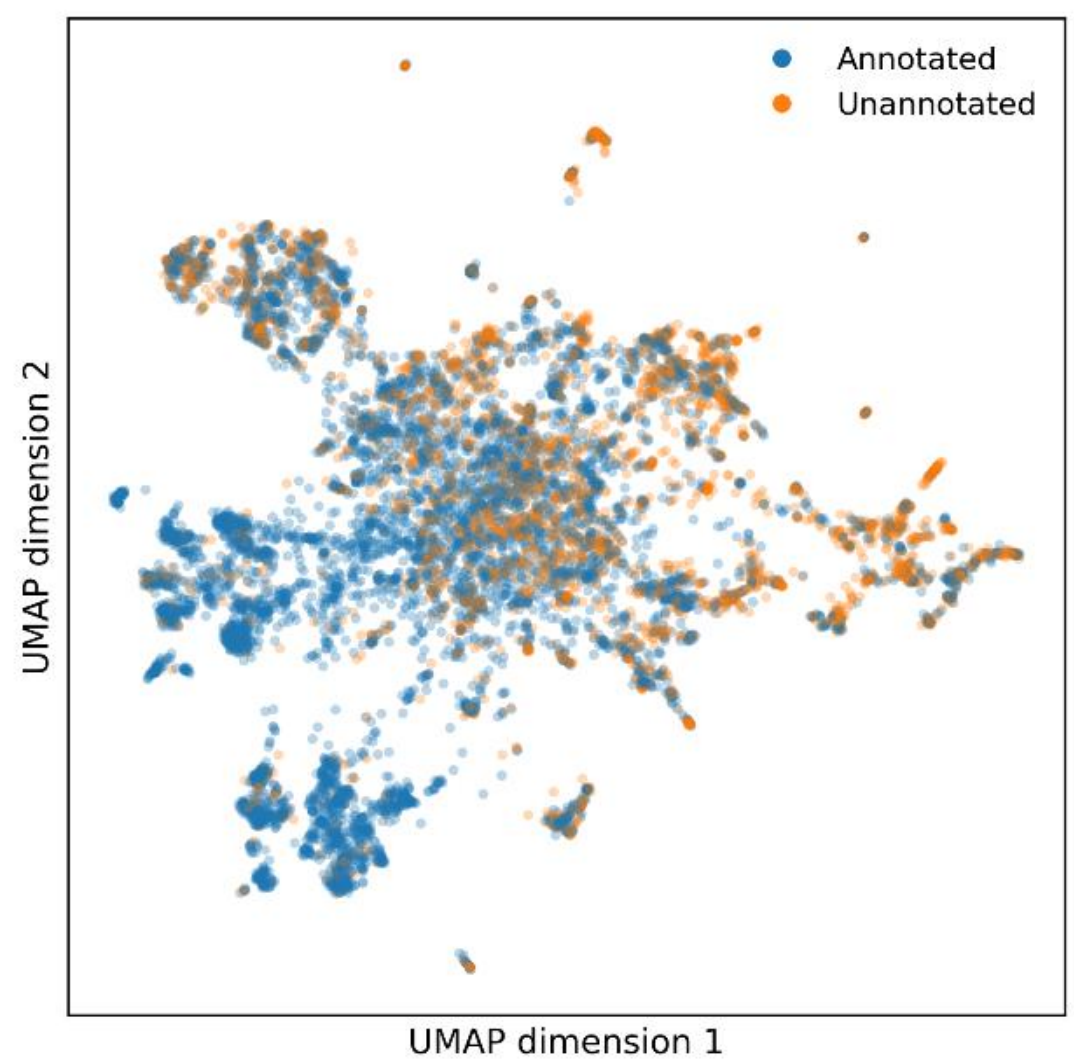

Supplementary Figure 1. Chemical-space distribution of compounds with and without resolved target annotations. UMAP visualization of the screening collection based on Morgan molecular fingerprints. Each point represents a compound structure and is colored according to whether a target annotation was resolved by SCOPE (annotated) or no target annotation was retrieved (unannotated). Annotated and unannotated compounds show extensive overlap across the UMAP space, with no clear global segregation by annotation status, indicating that the annotated subset broadly spans the chemical diversity represented in the screening collection.

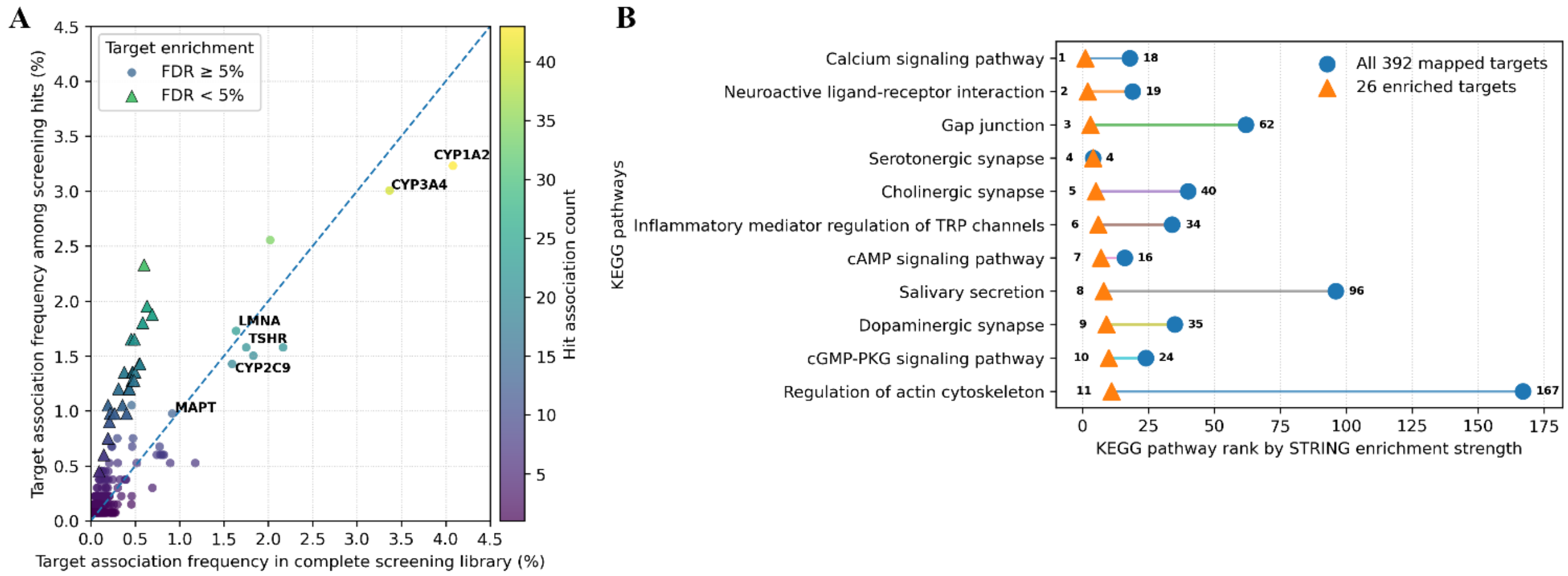

Supplementary Figure 2. Effect of screened-library background and target enrichment on target and pathway prioritization. (A) Comparison of target association frequency among screening hits with target association frequency in the complete screened library. Each point represents a mapped target, and its color indicates the number of hits associated with it, whereas the shape indicates target-enrichment significance at a 5% FDR cutoff. The dashed diagonal represents equal target representation among screening hits and the complete screened library, with targets above the diagonal relatively overrepresented among hits. Selected targets near the diagonal and highly represented library targets are labeled for illustration. **(B)** Comparison of KEGG pathway ranks obtained from STRING functional enrichment using all 392 mapped hit targets versus the 26 targets enriched at 5% FDR. Pathways are ranked by STRING enrichment strength. Numbers adjacent to the symbols indicate pathway rank within each analysis. Target enrichment substantially altered pathway prioritization, including elevation of the calcium signaling pathway from rank 18 among all mapped targets to rank 1 among the enriched targets.

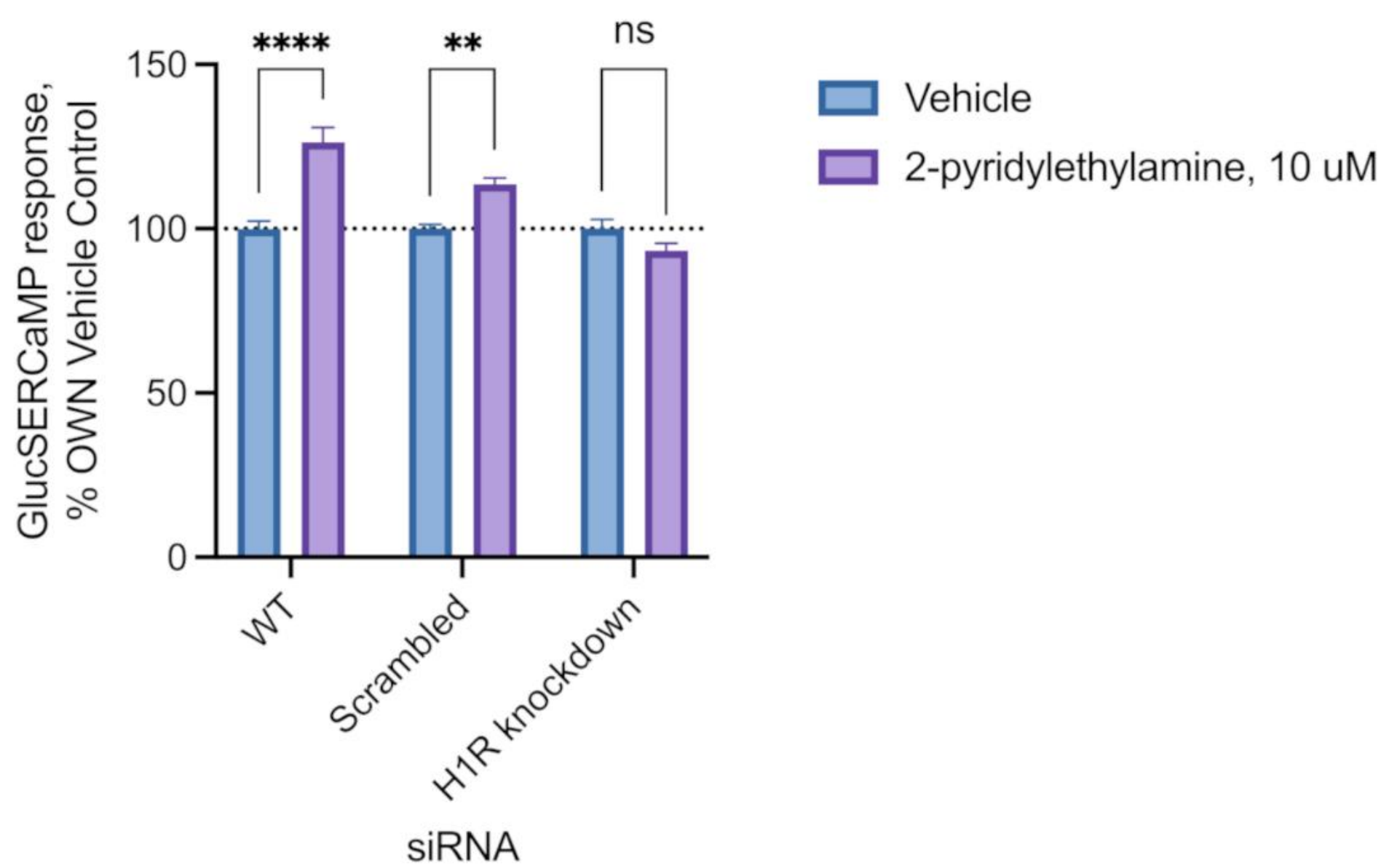

Supplementary Figure 3. siRNA-mediated knockdown of HRH1 reduces GLuc SERCaMP secretion. SH-SY5Y GLuc SERCaMP reporter cells were reverse transfected with HRH1-targeting siRNA or a non-targeting scrambled control. At 24 hours post-transfection cells were moved into medium containing 1.5% serum for 24 hours. Cells were then treated with 10 uM 2-pyridylethylamine, or vehicle control, for 16 hours. GLuc SERCaMP secretion was measured and normalized to vehicle control for each transfection condition. siRNA-mediated knockdown of HRH1 resulted in a significant reduction in GLuc SERCaMP response following 2-pyridylethylamine treatment, compared to wildtype and scrambled controls, indicating a role for HRH1 in regulating exodosis. Data here represents the results of five independent experiments ($n \geq 3$ per experiment) all normalized to their own vehicle control condition. (*<0.05, **<0.01, ****<0.0001 by two-way ANOVA).

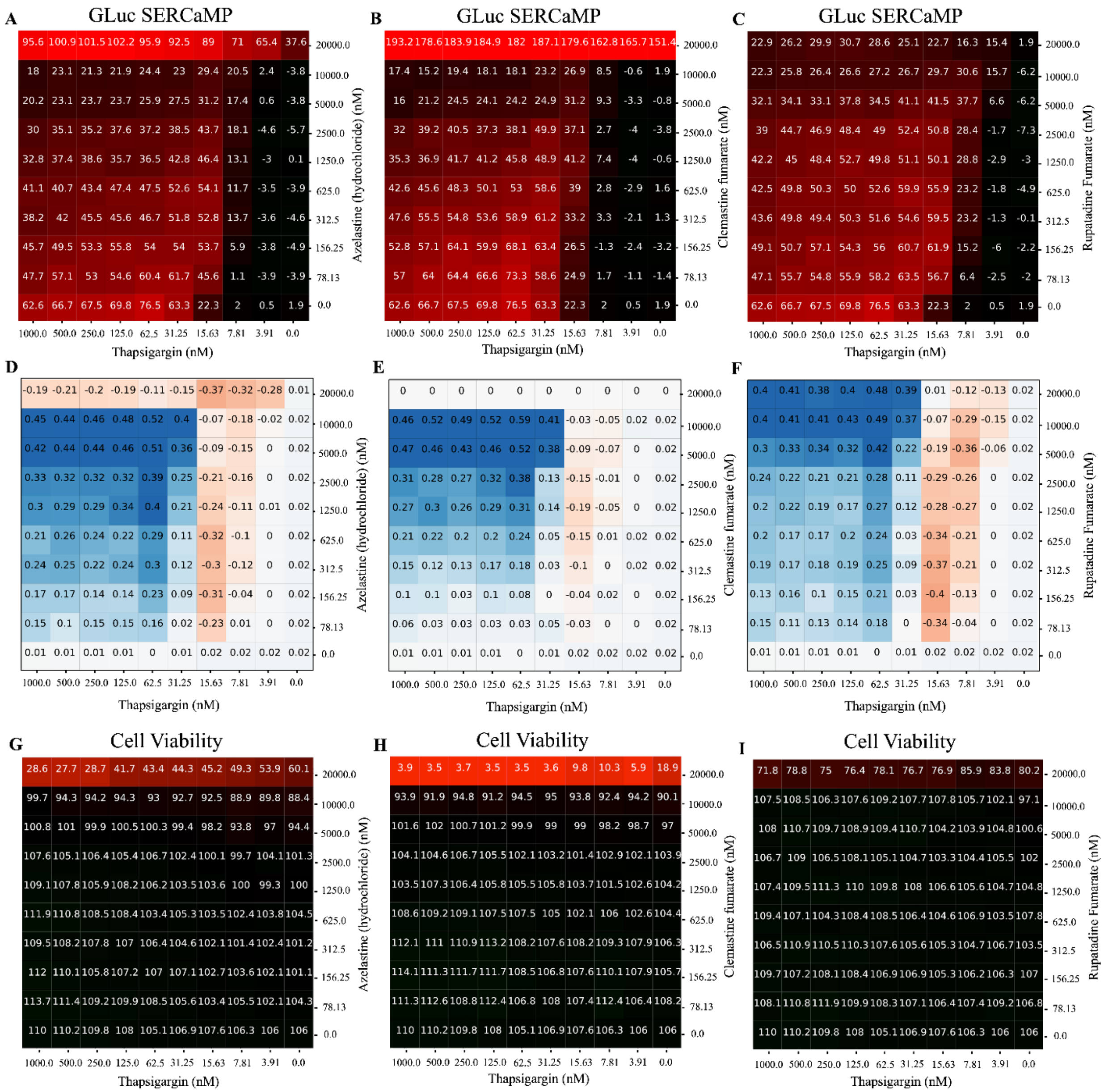

Supplementary Figure 4. Heat map plots of % GLuc SERCaMP response, delta Bliss scores, and cell viability for combination dose-response matrices for thapsigargin in combination with three compounds: azelastine hydrochloride, clemastine fumarate, and rupatadine fumarate. Panels (A,D,G) correspond to azelastine hydrochloride, panels (B,E,H) to clemastine fumarate, and panels (C,F,I) to rupatadine fumarate. The top row, panels (A-C), shows the GLuc SERCaMP response matrices, with thapsigargin concentration (nM) on the x-axis and partner compound concentration (nM) on the y-axis. Each cell represents the measured response at a given drug-dose pair. The middle row, panels (D-F), shows the corresponding delta Bliss synergy maps derived from the same dose matrices. Here, positive and negative values indicate antagonism and synergistic responses respectively. The bottom row, panels (G-I), shows the corresponding cell viability maps. Both, azelastine and clemastine, showed significant toxicity at the highest concentration tested (20 μM).
